# Research on the developmental patterns of the preschool children’s executive function under cross-ethnic background

**DOI:** 10.3389/fpsyg.2026.1984963

**Published:** 2026-10-29

**Authors:** Quan Li, Xu Deng, Xiaotong Li, Tingyong Feng

**Affiliations:** 1 College of Teacher Education, Qujing Normal University, Qujing, China; 2 Committee Organization Department, Qujing Normal University, Qujing, China; 3 School of Education and Psychological Sciences, Sichuan University of Science & Engineering, Zigong, China; 4 Faculty of Psychology, Southwest University, Chongqing, China

**Keywords:** 4–6 year olds, cross-ethnic background, developmental patterns, executive function, preschool children

## Abstract

Executive function (EF) is foundational for school readiness, mental health, and social adaptation. Although cross-national studies document cultural variation in EF, less is known about whether EF differs across ethnic groups within the same national and educational context. In China, Yi, Bai, and Naxi children may differ in home language, childrearing practices, and educational beliefs, making them informative for examining universal and context-specific patterns of EF development. This study examined Executive Function (EF) development in a sample of 190 Chinese ethnic children (Yi, Bai, Naxi; 115 boys, 75 girls) aged 4–6 years. These findings revealed that, (1) Overall EF scores were highest for Bai children, followed by Naxi children, and than Yi children. (2) For inhibition control (IC), at ages 4 and 5, Bai and Naxi children performed significantly better than Yi children, whereas no significant differences were observed among the three ethnic groups at age 6. (3) For cognitive flexibility (CF), Bai and Naxi children are significantly better than Yi children. On the size, color, and shape sub-dimensions, pass rates were highest for Bai children, followed by Naxi and then Yi children; on the quantity sub-dimension, pass rates were highest for Naxi children, followed by Bai and then Yi children. (4) For working memory (WM), at ages 4 and 6, the ranking of scores across the three ethnic groups showed that Bai children obtained the highest scores, followed by Naxi children and than Yi children; at age 5, Bai children scored significantly higher than Naxi and Yi children. These findings contribute to theories of EF development by revealing both age-related convergence and cross-ethnic variation, and they inform culturally responsive early childhood education and intervention.

## 1 Introduction

Executive function (EF), also referred to as executive control or cognitive control, encompasses a set of top-down, higher-order cognitive processes that coordinate and regulate thoughts, actions, and emotions in the service of goal-directed behavior. EF is considered fundamental to organized and adaptive human conduct (Diamond, 2013; Zelazo, 2015). Among these, inhibition control, working memory, and cognitive flexibility are the three core components of executive function (Sokol-Hessner et al., 2009). Executive function is the sum of the prefrontal brain functions that individuals employ when solving real-world problems. It represents one of the most important distinctions between humans and animals and is among the most remarkable achievements of the human brain in the course of evolution. In daily life, we rely on executive function to inhibit irrelevant stimuli, break away from habitual thinking, make decisions, and set priorities and sequences for our actions (Banich, 2009; Miyake and Friedman, 2012). Meanwhile, executive function is a critical skill for individuals’ physical and mental health as well as social adaptation, and it significantly predicts various aspects of individual outcomes, including externalizing behaviors (O’Toole et al., 2019; Wilson et al., 2018), academic achievement (Liu, 2019; Welsh et al., 2014), and mental health (Chavez-Arana et al., 2018; Zelazo, 2020).

With the advancement of globalization, research on executive function (EF) has received increasing attention. In particular, the study of EF in childhood—as a core component of cognitive development—has become an important area within developmental psychology. In an increasingly interconnected world, cross-cultural understanding and international collaboration are vital for scientific progress. Accordingly, there is a growing need for developmental research to adopt a cross-cultural perspective, engage with international scholarly discourse, and address the demands of diverse societies. To better understand the developmental mechanisms underlying children’s EF, researchers have previously conducted numerous cross-cultural studies across different cultural contexts, aiming to identify both universal and culturally specific patterns of EF development.

Prior work has documented substantial cross-cultural differences in executive function between North American and Asian children (Oh and Lewis, 2008; Sabbagh et al., 2006). Sabbagh et al. (2006) measured executive function and theory of mind in 109 Chinese and 107 American children, finding that Chinese children outperformed American children on executive function but lagged behind American children in theory of mind development. Yang et al. (2005) compared Chinese and American children and found that Chinese children were superior to American children in inhibitory control but did not show an advantage in working memory (Yang et al., 2005). Hou et al. (2023) examined children aged 4.52 years Zhuang and Han samples from Mainland China, and found that no difference in executive function and theory of mind link between two ethnic groups. Veraksa et al. (2024) compared executive function components in 51 Russian and 51 Japanese children aged 5–6.9 years, showing that Russian children exhibited higher levels of cognitive flexibility, whereas Japanese children showed higher levels of inhibition. In a more systematic comparison, 165 German and Iranian children aged 3–4 years were assessed on multiple cognitive tasks; results indicated that Iranian children outperformed German children in inhibitory control, while German children performed better in working memory, cognitive flexibility, and verbal fluency (Naderi et al., 2024; Nejati et al., 2024). Taken together, these findings suggested that the development of executive function (EF) exhibits significant differences across distinct cultural contexts. But it is unclear whether the differences also exist among different ethnic groups within the same broader cultural background.

China is a multi-ethnic country in which ethnic groups may differ in cultural practices, language ecologies, and educational experiences, all of which could shape children’s EF development (Doebel and Lillard, 2023). Comparing ethnic groups within a single national context offers a useful complement: because such groups share the same national language policy, educational system, and legal framework, between-group variation is more likely to reflect proximal mechanisms—home language ecology, childrearing practices, parental expectations, and the quality of local preschool provision—rather than macro-institutional differences. Existing evidence indicates that each of these mechanisms is associated with children’s EF development (Chen et al., 2025; Schirmbeck et al., 2020; Rea-Sandin et al., 2021). Examining whether, and at which ages, ethnic-group differences emerge within a shared national context can therefore help distinguish culturally patterned variation from variation attributable to national institutions. Existing work within China suggests age-related growth in EF across groups, while also indicating group-specific patterns in developmental timing (Hou et al., 2023; Li and Tan, 2023). Nevertheless, it remains unclear whether and how EF task progression and developmental timing differ across ethnic groups within the same broader national-cultural context. Therefore, the present study examines EF development amongYi, Bai, and Naxi preschool children aged 4–6 years.

### 1.1 Present study

The present study selected three representative ethnic groups in China (Yi children, Bai children, and Naxi children) as participants to examine the developmental patterns of executive function (EF) in children aged 4 to 6 years and to conduct a cross-group comparative study. All participants were recruited from township-level kindergartens with broadly comparable local economic development and teaching conditions. We note that ethnicity is confounded with contextual factors (e.g., region and language/schooling experiences) in this sampling frame; therefore, our analyses are intended to describe between-group differences rather than isolate ethnic or sub-cultural effects. The study employed general EF tasks, namely the Day/Night task, the Grass/Snow task, the Wisconsin Card Sorting Task (WCST), and the listening sentence span task. Two hypotheses were examined: (1) There are significant differences in EF development among the three ethnic groups; (2) The developmental trajectories of executive function (EF) components may differ across the three ethnic groups between the ages of 4 and 6.

## 2 Materials and methods

### 2.1 Participants

The study included a pilot experiment and a formal experiment. A total of 92 children from ethnic minorities at local kindergartens participated in the pilot experiment (e.g., 30 Yi children, 29 Bai children, and 33 Naxi children). The pilot experiment results showed that all children could understand the experimental materials and follow the experimenter’s instructions. Based on the pilot experiment, we refined the experimental materials and instructions. The formal experiment was conducted 3 months after the pilot. To ensure cultural appropriateness for all groups, the materials featured items commonly found in Yunnan, China, such as bird nests and dolls.

A total of 190 children aged 4 to 6 years were recruited from local kindergartens to participate in this study, including 115 boys and 75 girls. All participants were from ethnic minority groups residing in border regions of China, which are key ethnic autonomous areas supported by the local government. Notably, these children had no formal exposure to the Chinese language before entering kindergarten and received bilingual education (Chinese and their native language) thereafter. Detailed demographic information of the participants is presented in Tables 1, 2.

**Table 1:** Detailed information of the participants.

Ethnicity	Gender	4-years-old (48 ~ 54 months)	5-years-old (55 ~ 66 months)	6-years-old (67 ~ 78 months)
Yi children (*N* = 55)	Male: 31	8	12	11
	Female: 24	11	7	6
	Total: 55	19	19	17
Bai children (*N* = 65)	Male: 39	12	9	18
	Female: 26	9	10	7
	Total: 65	21	19	25
Naxi children (*N* = 70)	Male: 45	10	19	16
	Female: 25	10	5	10
	Total: 70	20	24	26

**Table 2:** Detailed demographic information.

Groups	Age at K entry	Length of K attendance	Home language	Parental education	Occupation
Bai	3-years-old	7:40 a.m. to 5:20 p.m.	Bai Language	Junior high school education or below	Agriculture
Naxi	3-years-old	7:40 a.m. to 5:20 p.m.	Naxi Language	Junior high school education or below	Agriculture
Yi	3-years-old	7:40 a.m. to 5:20 p.m.	Yi Language	Junior high school education or below	Agriculture

An *a priori* power analysis was conducted in G*Power 3.1 (Faul et al., 2007) for the 3 × 3 × 2 between-subjects design, using the F-test for fixed-effects ANOVA (special, main effects and interactions) with *α* = 0 0.05 and power = 0.80. Assuming a medium effect (*f* = 0.25; Cohen, 1988), the required total sample size was 158 for the main effects of ethnicity and age (numerator df = 2) and 197 for the ethnicity × age interaction (numerator df = 4). The obtained sample of 190 children therefore exceeded the requirement for the main effects but fell marginally short — by 7 participants (3.6%) — of that for the ethnicity × age interaction, corresponding to achieved power of 0.87 and 0.79, respectively. It should be noted, however, that the effects actually observed (η^2^ = 0.095–0.360) exceeded this threshold, indicating that the analyses we report were adequately powered; the shortfall concerns only effects at or near the sensitivity limit of the design.

### 2.2 Design

The present study employed a 3 (ethnic groups: Yi children, Bai children, Naxi children) × 3 (age: 4-years-old, 5-years-old, 6-years-old) × 2 (sex: boys VS girls) between-subjects design‌. Ethnic groups’ children who come from the representative nationalities in Yunnan province, China. The study primarily examined the developmental patterns of executive function among the three ethnic groups, aiming to uncover the underlying reasons for the developmental patterns of executive function in these children.

### 2.3 Procedure

After getting the consent from the parents and kindergarten’s permission, children were tested individually in a quiet room. The testing was conducted by two experimenters: one was a professional graduate student majoring in psychology, and the other was an assistant experimenter, a local kindergarten teacher fluent in the ethnic minority language. As a warm-up, a 5–10 min session of non-test-related play and conversation among the child, experimenter, and assistant was conducted in the classroom to minimize anxiety and promote cooperation. The experimenters then assessed the children’s executive function (EF) using three tasks. Notably, the three tasks were administered in a randomized order, and the scores from each task were summed to calculate the total EF score for each child. Specifically, a computer-based random number generator was used to create a unique random sequence of the three tasks (order 1 to 3) for each child. The experimenter presented the tasks according to this predetermined sequence for each participant.

### 2.4 Measures

Children’s executive function was assessed in the present study using the Day/Night task, the Grass/Snow task, the Wisconsin Card Sorting Task (WCST), and the Listening Sentence Span task. All testing was conducted by two experimenters: a graduate student majoring in psychology and an assistant experimenter—a local kindergarten teacher who had used the target ethnic language as the primary language of instruction for at least 5 years and who self-rated her fluency in both the target ethnic language and Mandarin as 4.5 or above on a 5-point scale.

#### 2.4.1 Task 1: day/night and grass/snow

The Day/Night and Grass/Snow tasks are used to measure children’s inhibitory control (Simpson and Riggs, 2005; Simpson and Riggs, 2009).

Before the formal experiment, the experimenter presented four pictures to the children one by one and asked them to identify the content of each picture. After ensuring that the children responded correctly, the experimenter instructed them as follows: when the picture of the “sun” was shown, they should answer “night”; when the picture of the “moon and stars” was shown, they should answer “day”; when the picture of “grass” was shown, they should answer “snow”; and when the picture of “snow” was shown, they should answer “grass.” Each of the four stimuli (sun, moon and stars, grass, snow) was presented four times in a random order. The child received one point for each correct answer, and the final score of children’s inhibition control was calculated by the sum up all sets together (Figure 1).

**Figure 1:**
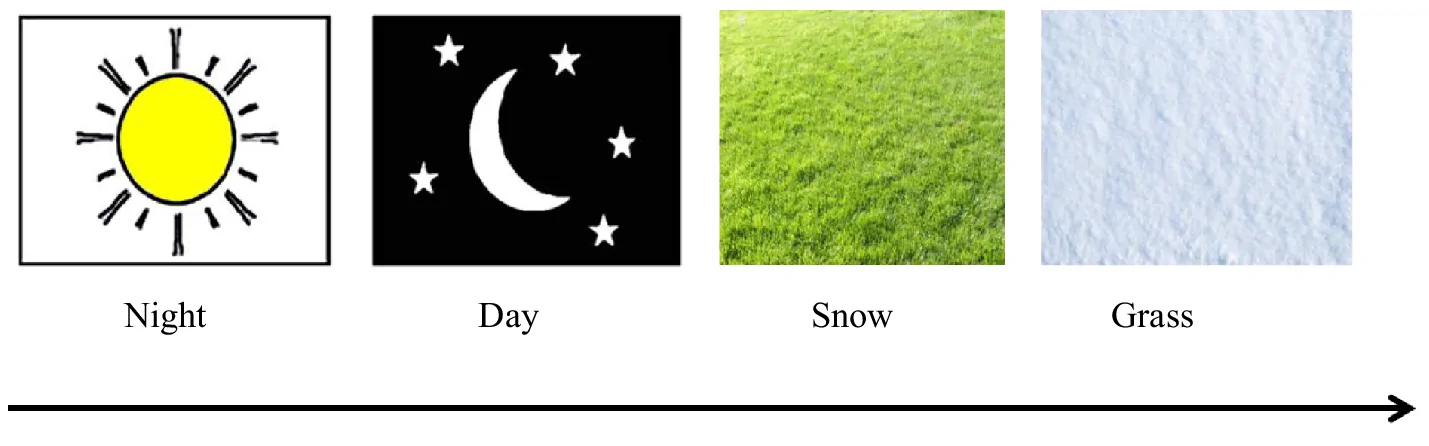
Schematic diagram of the inhibitory control task.

#### 2.4.2 Task 2: Wisconsin card sorting task

The Wisconsin Card Sorting Task (WCST) is used to measure children’s cognitive flexibility (Kaland et al., 2008). This task requires children to inhibit the previously dominant rule and sort cards according to a new rule. As shown in Figure 2, children are first asked to sort cards by color. After they fully understand the rule and have completed eight correct sorts, they are then required to inhibit the previously dominant rule and sort by a new rule—shape. During the sorting process, the experimenter records the number of correct and incorrect sorts. The number of correct sorts is used to determine whether the child is able to inhibit the previous rule and discover the new rule. In this study, a total of 102 cards were used: color (32 cards), shape (32 cards), number (32 cards), and size (16 cards). The size category served as a practice set, with a passing criterion of 12 correct sorts. The other three categories served as the experimental sets. For each dimension, a score of 20 or more correct sorts (out of 32) was counted as a pass (coded as “1”), and a score below 20 correct sorts was coded as “0.” The final score for children’s cognitive flexibility was calculated by summing the scores across the three dimensions.

**Figure 2:**
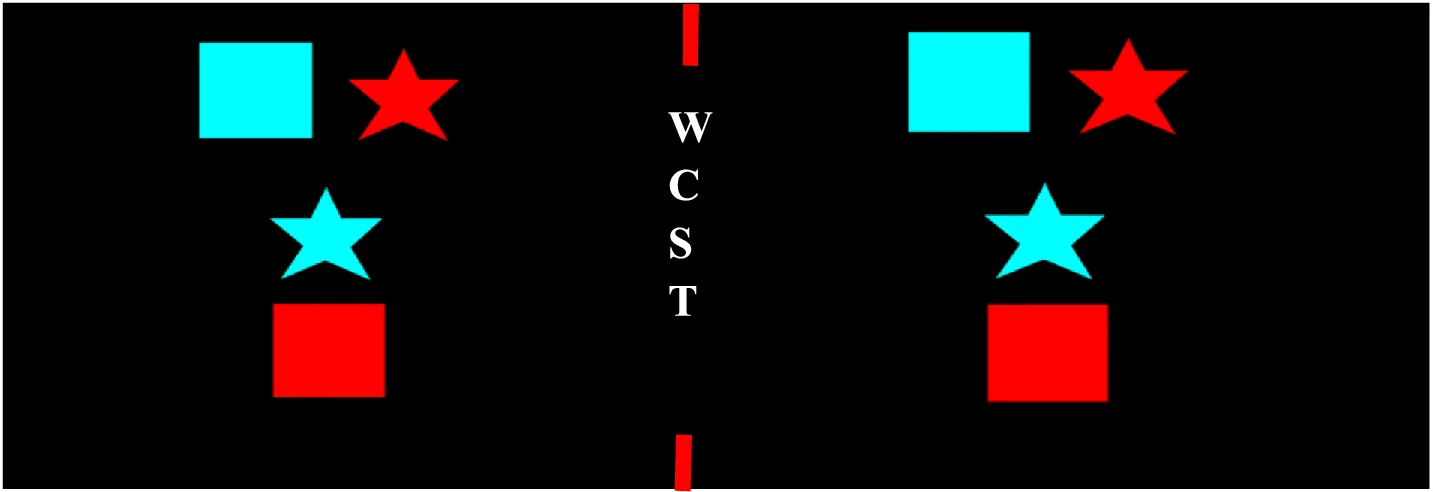
Schematic diagram of the cognitive flexibility task.

#### 2.4.3 Task3: listening sentence span task

The Listening Sentence Span Task is used to measure children’s working memory development (Duff and Logie, 2001). The task consists of two parts. The first part requires children to judge whether each sentence is true or false, which primarily assesses their general knowledge and comprehension. The second part mainly measures children’s ability to process and refresh sentence information in memory. The entire task (see Table 3) includes two-sentence series, three-sentence series, and four-sentence series. Except for the four-sentence series, each series contains two sets of sentences. For each set, children must correctly judge the truth value of each sentence and also remember the last word (or character) of each sentence to pass that set. In this study, a pass was coded as “1” and a fail as “0.” The final score for children’s working memory was calculated by summing the scores across the four sentence series.

**Table 3:** Materials for the listening sentence span task.

Sentence		True/False	Answer
Two-sentence series	1. Students go to school	True	school
	2. The kitten (animal) is covered with fur	True	fur
	1. I wash my face with warm water	True	wash face
	2. I have ten toes on my hand	False	toes
Three-sentence series	1. The grass grows indoors	False	indoors
	2. The tree is full of leaves	True	leaves
	3. The bird has its own nest	True	nest
	1. I watch TV at home	True	watch TV
	2. There is a doll in the cradle	True	doll
	3. The sun appears at night	False	night
Four-sentence series	1. The teacher teaches us to learn	True	learn
	2. The students are playing basketball on the mountain	False	basketball
	3. Drink water with chopsticks	False	drink water
	4. The gardener is mowing the grass	True	mow the grass

## 3 Results

To delve deeper into the developmental patterns of the three ethnic groups, we analyzed the development patterns of children’s EF for each sub-EF, focusing separately on the three ethnic groups. Data were analyzed using SPSS 28. To address *post hoc* multiple comparisons within each ANOVA, we employed Bonferroni-adjusted *p* values, and all reported *p* values are the adjusted values.

### 3.1 Developmental patterns on executive function among three ethnic groups

Scores for inhibition control, cognitive flexibility, and working memory were converted into Z-scores and then summed to create a new composite variable labeled “executive function (EF).” The EF scores of the three ethnic groups, from highest to lowest, were Bai, Naxi, and Yi. As can be seen from Figure 3, To investigate the differences in EF across ethnic groups and ages, we performed 3 (group: Yi, Naxi and Bai) × 3 (age: 4 years old, 5 years old and 6 years old) ANOVA. The results revealed that the ethnicity main effect was significant, *F_(2,172)_* = 22.471, p < 0.001, η^2^_p_ = 0.208; the age main effect was significant, *F*_(*2,172*)_ = 10.955, *p* < 0.001, η^2^_p_ = 0.114; the gender main effect was not significant, *F*_(*1*,*172*)_ = 0.002, *p* > 0.05, η^2^_p_ = 0.000; the interaction between ethnicity and age was significant, *F*_(*4,172*)_ = 12.074, *p* < 0.01, η^2^_p_ = 0.095; The interaction between ethnicity and gender was also not significant, *F*_(*2,172*)_ = 2.797, *p* > 0.05, η^2^_p_ = 0.000. And the interaction between gender and age was not significant, *F*_(*2,172*)_ = 0.638, *p* > 0.05, η^2^_p_ = 0.001. Multiple comparisons on the ethnicity variable revealed that Bai children scored significantly higher than Naxi, and Yi children, *ps*<0.05.

**Figure 3:**
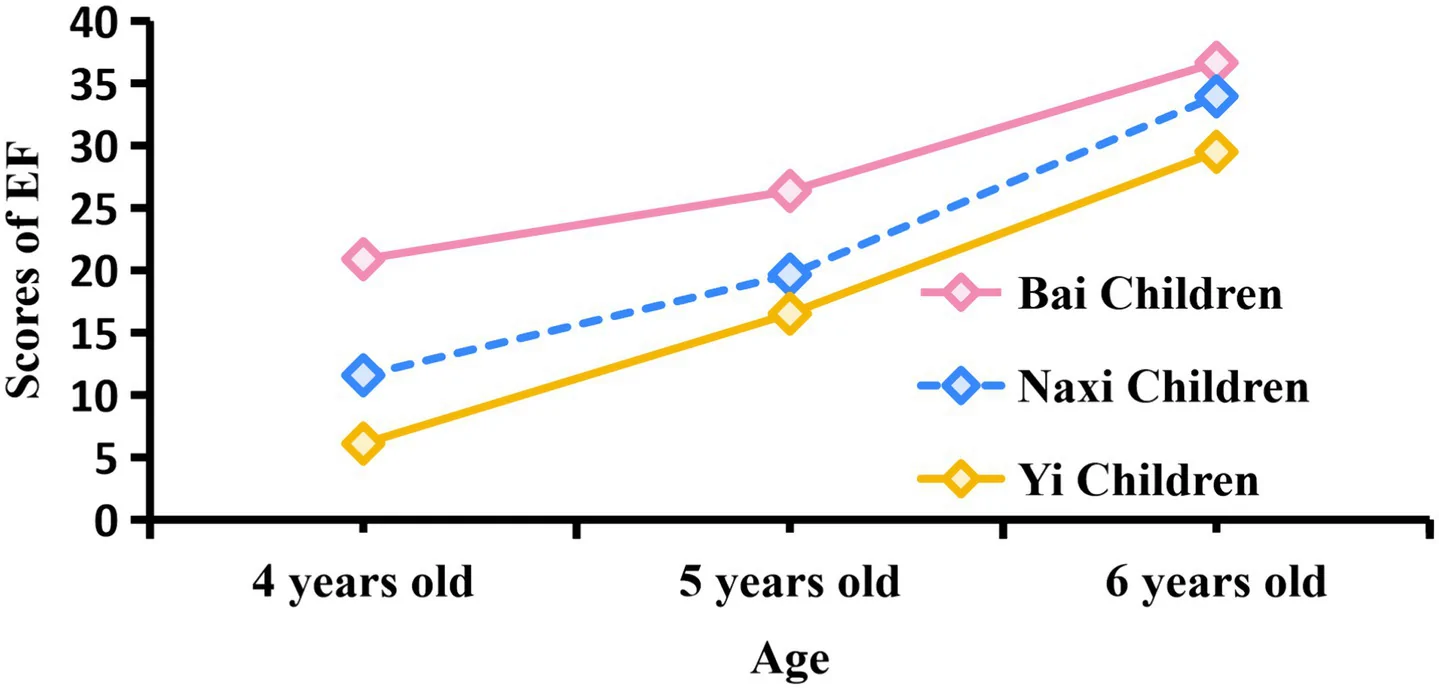
Comparison of EF scores among the three ethnic groups.

We performed a simple effect test of ethnicity within a constant ethnic group. For the 4-year-old group, there was no significant difference in EF scores between Bai and Naxi children, *p* > 0.05, whereas Bai children scored significantly higher than Yi children, △Mean = 1.877, *p* < 0.001, and Naxi children also scored significantly higher than Yi children, △Mean = 2.194, *p* < 0.001. For the 5-year-old group, Bai children scored significantly higher than Naxi children, △Mean = 1.455, *p* < 0.01, and also significantly higher than Yi children, △Mean = 1.400, *p* < 0.05; however, no significant difference was found between Naxi and Yi children, *p* > 0.05. For the 6-year-old group, significant differences in EF scores were observed among all three ethnic groups, *ps*< 0.05.

We performed a simple effect test of age within a constant ethnic group. For Bai children, no significant age difference was found between the 4-and 5-year-old groups, *p* > 0.05, but significant differences were found between the 5-and 6-year-old groups, △Mean = −1.945, *p* < 0.001, and between the 4-and 6-year-old groups, △Mean = 2.297, *p* < 0.01. For Naxi children, significant age differences were found among the 4-to 6-year-old, *ps*<0.01. For Yi children, no significant age difference was found between the 4- and 5-year-old groups, *p* > 0.05; a significant difference was found between the 4- and 6-year-old groups, △Mean = −1.209, *p* < 0.05; and no significant difference was found between the 5- and 6-year-old groups, *p* > 0.05.

### 3.2 Developmental patterns on inhibition control among three ethnic groups

As shown in Figure 4, the inhibition control scores of the three ethnic groups, from highest to lowest, were Bai children (M = 13.63, SD = 4.93), Naxi children (M = 11.26, SD = 7.41), and Yi children (M = 9.44, SD = 6.42). To examine the impact of ethnicity and age on the development of children’s IC, we conducted 3 (group: Yi, Naxi and Bai) × 3 (age: 4 years old, 5 years old and 6 years old) ANOVA. The results revealed that the main effect of ethnicity was significant, *F*_(*2,172*)_ = 23.23, *p* < 0.001, η^2^_p_ = 0.178. The main effect of age was significant, *F*_(*2,172*)_ = 395.89, *p* < 0.001, η^2^_p_ = 0.786; the ethnicity × age interaction was also significant, *F*_(*4*,*172*)_ = 8.019, *p* < 0.001, η^2^_p_ = 0.121. *Post hoc* comparisons indicated that Bai children differed significantly from Naxi children, △Mean = 0.805, *p* < 0.01, and Bai children differed significantly from Yi children, △Mean = 2.128, *p* < 0.001, whereas no significant difference was found between Naxi and Yi children, *p* > 0.05.

**Figure 4:**
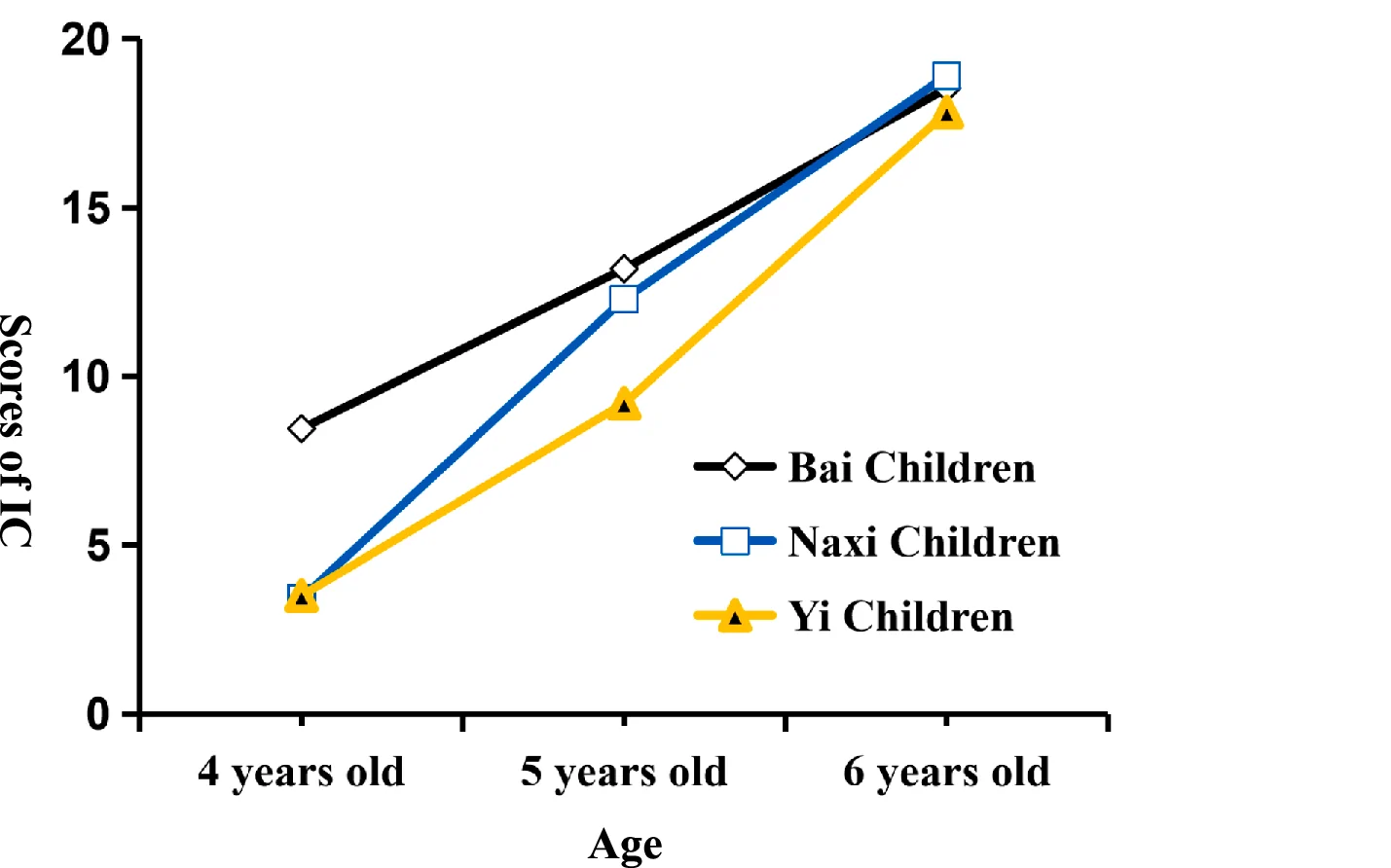
The development trend of IC among three ethnic groups.

With ethnicity held constant, a simple effect analysis was performed, focusing on the dimension of age. For Bai children, there were significant age differences in inhibitory control scores among the 4-, 5-, and 6-year-old groups, *ps*<0.001; for Naxi children, there were significant age differences among the three age groups, *ps* <0.001; and for Yi children, there were also significant age differences among the three age groups, *ps*<0.001.

Within each age group, simple effects of ethnicity were examined. For the 4-year-old group, the difference in inhibition control scores between Bai and Yi children was highly significant, △Mean = 1.772, *p* < 0.001, and the difference between Bai and Naxi children was also highly significant, △Mean = 1.642, *p* < 0.001, whereas no significant difference was found between Naxi and Yi children, *p* > 0.05. For the 5-year-old group, there was no significant difference between Bai and Naxi children, *p* > 0.05, Bai children performed significantly better than Yi children, △Mean = 1.156, *p* < 0.01, and significant difference was found between Naxi and Yi children, △Mean = 1.278, *p <* 0.05. For the 6-year-old group, no significant differences in inhibitory control scores were observed among Yi, Bai, and Naxi children, *ps* >0.05. Overall, at ages 4 and 5, Bai and Naxi children performed significantly better than Yi children in inhibitory control, whereas at age 6, no significant differences were found among the three ethnic groups, indicating that all groups had reached a relatively stable developmental pattern.

### 3.3 Developmental patterns on cognitive flexibility among three ethnic groups

To examine the impact of ethnicity and age on the development of children’s cognitive flexibility, we conducted 3 (group: Yi, Naxi and Bai) × 3 (age:4 years old, 5 years old and 6 years old) ANOVA (See Figure 5). The results showed that the main effect of ethnicity was not significant, *F*_(*2,172*)_ = 0.324, *p* > 0.05, η^2^_p_ = 0.004; the main effect of age was significant, *F*_(*2,172*)_ = 6.544, *p* < 0.001, η^2^_p_ = 0.071; and the ethnicity × age interaction was significant, *F*_(*4,172*)_ = 10.485, *p* < 0.001, η^2^_p_ = 0.196.

**Figure 5:**
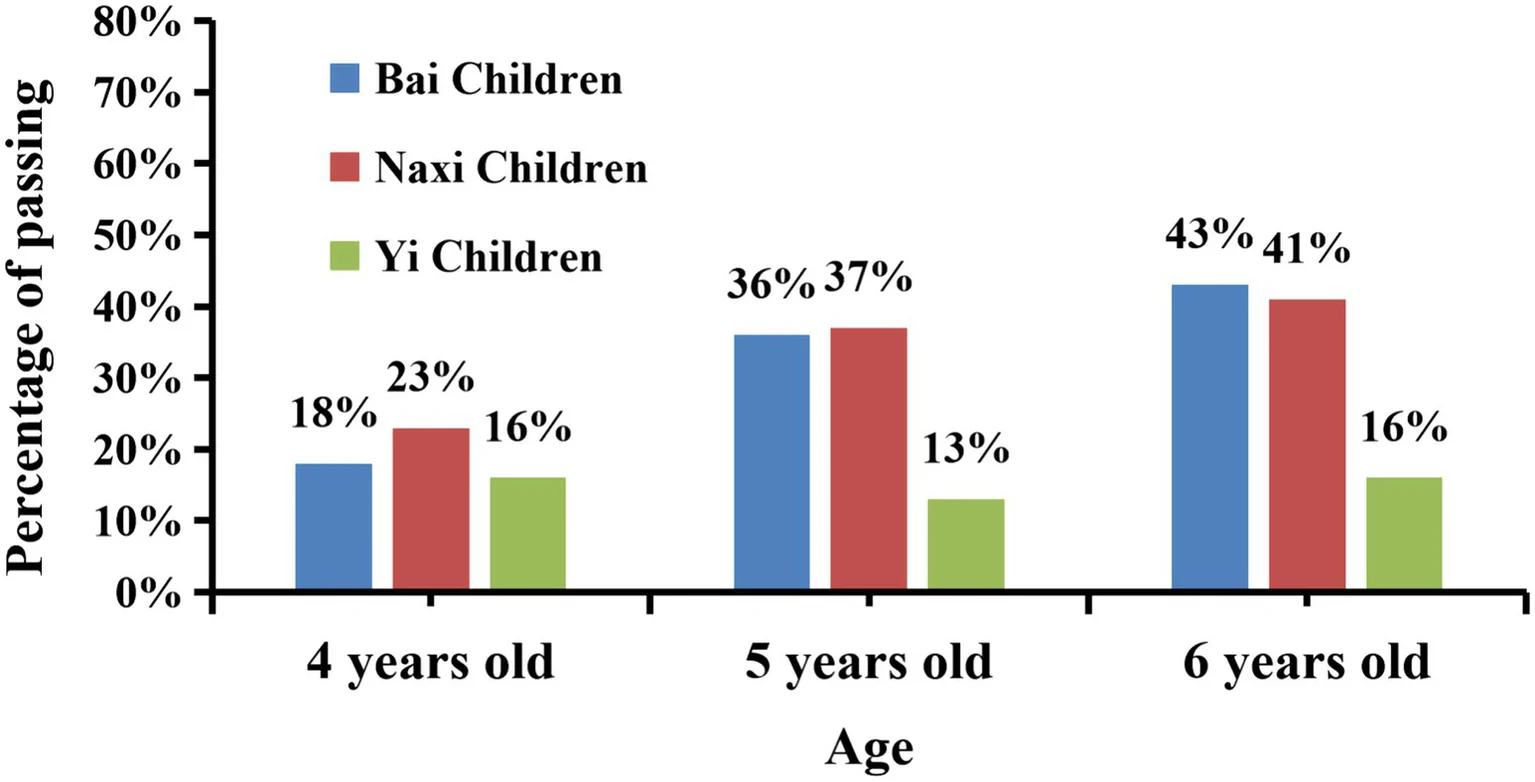
The percentage of WCST among three ethnic groups.

With age held constant, a simple effect analysis was conducted, examining ethnicity differences. For the 4-year-old group, Naxi children scored significantly higher than Yi children in cognitive flexibility, △Mean = 1.367, *p* < 0.05, whereas no significant differences were found between Bai and Naxi children or between Bai and Yi children, *ps*>0.05. For the 5-year-old group, both Bai and Naxi children scored significantly higher than Yi children, *ps*<0.001; For the 6-year-old group, no significant difference was found between Bai and Naxi children, *p* > 0.05, and both Bai and Naxi children scored significantly higher than Yi children, *ps*<0.05.

With ethnicity held constant, a simple effect analysis was conducted, examining age differences. For Bai children, no significant age differences were found among the 4-year-old, 5-year-old, and 6-year-old groups, *ps*>0.05. For Naxi children, the 4-year-old group differed significantly from the 6-year-old group, △Mean = 1.948, *p* < 0.001, and from the 5-year-old group, △Mean = 1.439, *p* < 0.01, whereas no significant difference was observed between the 5-and 6-year-old groups, *p* > 0.05. For Yi children, significant difference was found between the 4-and 6-year-old groups, *p* < 0.05; however, the 4-year-old group differed significantly from the 5-year-old group, △Mean = 1.750, *p* < 0.01, and the 5-year-old group also differed significantly from the 6-year-old group, △Mean = −2.790, *p* < 0.01. In summary, children aged 4 to 6 years showed significant age differences in cognitive flexibility. Specifically, for Naxi children, the 6-year-old group performed significantly better than the 4-year-old group; for Yi children, the 5-year-old group scored significantly higher than both the 4-and 6-year-old groups.

To further examine differences in cognitive flexibility among the three ethnic groups across the four sub-dimensions (size, quantity, color, shape), chi-square tests were conducted with ethnicity as the independent variable and each sub-dimension as the dependent variable. The results showed significant differences among Yi, Bai, and Naxi children on the sub-size dimension, χ^2^_(2)_ = 30.952, *p* < 0.001; on the sub-quantity dimension, χ^2^_(2)_ = 15.388, *p* < 0.001; on the sub-color dimension, a marginally significant difference was observed, χ^2^_(2)_ = 6.504, *p* = 0.065; and on the sub-shape dimension, the difference was significant, χ^2^_(2)_ = 18.934, *p* < 0.001.

Overall, for children aged 4 to 6 years, the pass rates on the cognitive flexibility tasks among the Bai, Naxi, and Yi groups showed the following rank order: on the size subdimension, the Bai group was the highest, followed by the Naxi group, with the Yi group the lowest; on the quantity subdimension, the Naxi group was the highest, followed by the Bai group, with the Yi group the lowest; on both the color and shape subdimensions, the Bai group was the highest, followed by the Naxi group, with the Yi group the lowest.

### 3.4 Developmental patterns on working memory among three ethnic groups

As shown in Figure 6, the working memory scores of the three ethnic groups, from highest to lowest, were Bai children (M = 1.58, SD = 1.380), Naxi children (M = 0.32, SD = 0.773), and Yi children (M = 0.23, SD = 0.641). To examine the impact of ethnicity and age on the development of children’s Working Memory, we conducted 3 (group: Yi, Naxi and Bai) × 3 (age: 4 years old, 5 years old and 6 years old) ANOVA. The results revealed that the main effect of ethnicity was significant, *F*_(*2,172*)_ = 51.922, *p* < 0.001, η^2^_p_ = 0.360; the age main effect was significant, *F*_(*2,172*)_ = 31.563, *p* < 0.001, η^2^_p_ = 0.254; the interaction between ethnicity and age was also significant, *F*_(*4,172*)_ = 6.620, *p* < 0.001, η^2^_p_ = 0.125.

**Figure 6:**
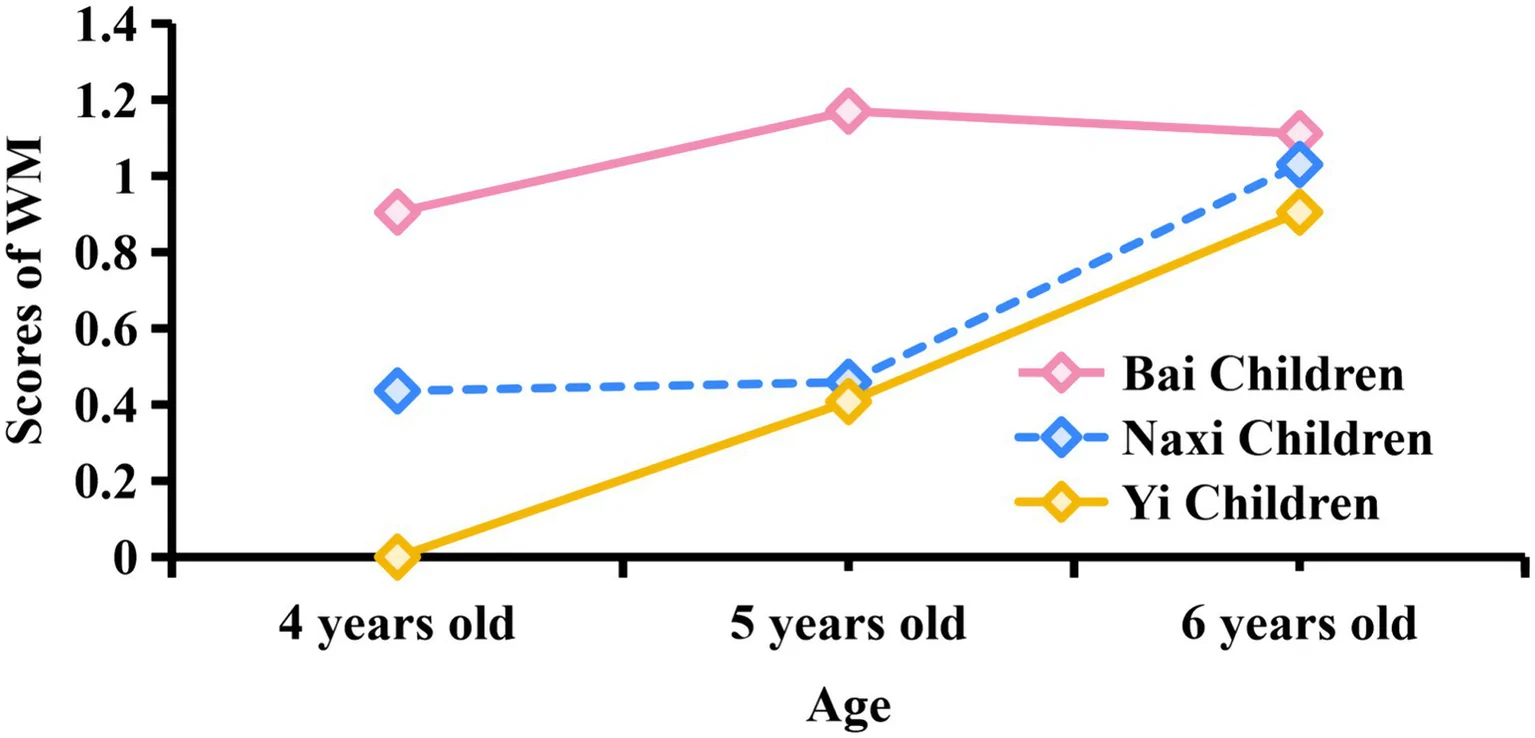
The development trend of WM among three ethnic groups.

With ethnicity held constant, a simple effect analysis was performed, focusing on the dimension of age. Among Bai children, significant age differences in working memory were observed among the 4-, 5-, and 6-year-old groups, *p* < 0.001. For Naxi children, no significant age differences were found between the 4-and 5-year-old groups, *p* > 0.05, whereas the 6-year-old group scored significantly higher than both the 4-and 5-year-old groups, *p* < 0.05. For Yi children, no significant age differences were found between the 4- and 5-year-old groups, *p* > 0.05, whereas the 6-year-old group performed significantly better than the 4-and 5-year-old groups, *p* < 0.05.

With age held constant, a simple effect analysis was performed, focusing on the dimension of ethnicity. For the 4-year-old group, significant differences were found between Bai and Naxi children, *p* < 0.01, with Bai children performing significantly better than Yi children, *p* < 0.001 and Naxi children also performing significantly better than Yi children, *p* < 0.001. For the 5-year-old group, significant differences were found between Bai and Naxi children, *p* < 0.01, with Bai children performing significantly better than Yi children, *p* < 0.01, whereas no significant difference was observed between Naxi and Yi children, *p* > 0.05. For the 6-year-old group, significant differences were found between Bai and Naxi children, *p* < 0.05, with Bai children performing significantly better than Yi children, *p* < 0.001, and Naxi children also performing significantly better than Yi children, *p* < 0.01.

## 4 Discussion

### 4.1 Development patterns of executive function

Overall, the three ethnic groups demonstrated age-related improvements across all executive function (EF) components, which is in line with the general developmental trajectory of EF during early childhood (Diamond, 2013; Zelazo, 2015). However, significant inter-group differences were observed. Bai children consistently outperformed both Naxi and Yi children in terms of the composite EF score, as well as in inhibitory control (IC) and working memory (WM). Naxi children generally exhibited intermediate performance levels, whereas Yi children scored relatively lower, particularly at the ages of 4 and 5. Notably, by the age of 6, ethnic differences in inhibition control diminished or even disappeared, indicating a convergence effect: all groups reached comparable levels. Schirmbeck et al. (2020) examined 170 children in Grades 2 and 4 from Hong Kong (*N* = 80) and Germany (*N* = 90) and found no significant differences in EF performance between the two groups at primary school age. This pattern is consistent with the notion that basic inhibitory processes may be less influenced by cultural variations compared to more complex EF components, such as cognitive flexibility (CF) and working memory (WM) (Doebel and Lillard, 2023). The overall mean scores of cognitive flexibility (CF) did not differ significantly among the three ethnic groups. However, significant interactions involving age and sub-dimensions (size, quantity, color, shape) revealed nuanced patterns. Naxi children demonstrated accelerated development in CF between the ages of 4 and 6, while Yi children showed a peak in CF performance at age 5. These findings support the notion that different sub-components of executive function (EF) follow distinct developmental trajectories and are differentially influenced by cultural contexts (Anderson, 2008; Kuwabara et al., 2011).

### 4.2 Possible cultural and educational correlates

Different natural environments, socio-historical backgrounds, and educational beliefs shape the unique thinking styles, personality traits, and psychological development of each ethnic group (Vygotsky, 1978; Berry et al., 2011; Rogoff, 2003; Kitayama and Cohen, 2010). Kuwabara et al. (2011) found that cultural differences already emerge in children as young as 4 to 5 years old, suggesting that child development is shaped not only by individual characteristics but also by cultural contexts (Lewis et al., 2009; Zhou et al., 2009). The Bai ethnic group is known for its inclusiveness, readily embracing diverse cultures and adopting external elements that benefit its own development (Zhao, 2009). This may provide a supportive context for children’s education; early exposure to multicultural influences among Bai children could be associated with the development of self-regulation, cognitive flexibility, and working memory. However, these constructs were not directly measured, and this interpretation remains speculative. These findings tentatively suggest that, in the present study, the executive function of Bai children aged 4–6 years may have been temporarily superior to that of Yi and Naxi children. During the working memory assessment, children from the three ethnic groups mentioned animals from their respective totems: most Bai children mentioned “chicken, tiger…,” Naxi children mentioned “frog…,” and Yi children mentioned “tiger…,” which may reflect their unique totemic cultures (Du, 2008; He, 1991; Jiao et al., 2014).

Previous research has demonstrated that children’s everyday psychological abilities are closely related to their environments (Liefländer et al., 2013). Hugh Matthews and colleagues compared rural and urban children in the United Kingdom and found a temporary “dislocation” in emotional development: rural children attributed more positive futures to rural life, whereas urban children perceived rural areas as small, remote, and poor. This is consistent with the possibility that children’s environments could exert lasting effects on their early psychological development (Matthews et al., 2000). Studies have also shown that the period from 2 to 5 years of age is a stage of rapid executive function development (Li and Wang, 2004). The Yi, Bai, and Naxi ethnic groups each have their own living environments and cultures, which may shape their children’s unique executive function profiles and thereby help account for the observed differences in executive function development among 4- to 6-year-old Yi, Bai, and Naxi children.

The Bai ethnic group places great emphasis on early childhood education, particularly within the family, which may reflect distinctive ethnic characteristics. As a Bai proverb states, “A good dragon-head sanxian produces good songs and tunes; a good parent family produces good sons and daughters”. Family education may play a crucial role throughout children’s executive function development (Zhang, 2008). This could be one potential factor that helps explain the temporarily superior executive function development observed among Bai children relative to Yi and Naxi children. Furthermore, from the perspective of inhibitory control development, the three ethnic groups showed consistency in executive function development, with performance ranking as Bai > Naxi > Yi. This commonality is consistent with the hypothesis that the cultural identity formed during the development of minority cultures helps reduce differences in living environments among minority children and strengthen intra-ethnic connection—though this interpretation remains tentative (Zheng and Ma, 2010). Nevertheless, the three ethnic groups also exhibited differences in inhibition control development: at ages 4 and 5, Bai and Naxi children outperformed Yi children, whereas at age 6, no significant differences were observed among the three groups, indicating that inhibitory control had largely matured by age 6 across all groups. The development of inhibitory control is more strongly governed by neurological maturation; accordingly, the developmental trajectories of different groups tend to converge once maturation is complete (Diamond, 2013; Garon et al., 2008). This is consistent with the present findings, in which ethnic differences in inhibitory control had disappeared by age 6. By contrast, the development of working memory and cognitive flexibility is more dependent on experiential input from cultural practices (e.g., language environments and schooling). Because such differences accumulate over time, they tend to be more enduring (Best and Miller, 2010; Carlson, 2005). This may explain why the differences in WM and CF observed in the present study persisted through age 6.

However, several alternative explanations should be considered. First, although the three groups were recruited from township kindergartens with comparable schedules and broadly similar parental education and occupation profiles (Table 2), we did not measure household income or home learning environment, and subtle socioeconomic differences cannot be ruled out. Second, all children were heritage-language speakers receiving bilingual instruction; differences in Mandarin proficiency—which we did not directly assess—could influence performance on verbally mediated tasks. Third, differences in preschool quality, teacher qualifications, and parental involvement in education may contribute independently of ethnic identification. Ethnicity may be correlated with these contextual factors in our sampling frame; the present design cannot determine which of them accounts for the observed differences.

In summary, the present study not only enriches the field of early childhood executive function development but may also provide a potential scientific basis for promoting healthy child development and formulating educational intervention strategies. Since the 1990s, when Lyon and Krasnegor began investigating children’s prefrontal functions, cognitive control, and self-regulation, research on children’s executive function has grown considerably, both in China and internationally. Existing studies have suggested that children’s executive function can be improved through practical interventions (Bai and Chun, 2011). However, not all intervention programs are equally effective; only those tailored to children’s developmental characteristics are likely to yield improvements, whereas inappropriate interventions may increase children’s burden without achieving the desired results (Diamond and Lee, 2011). Therefore, grounded in practical research, this study employed a standard executive function paradigm to conduct field investigations of executive function in Yi, Bai, and Naxi children aged 4 to 6 years. The findings may provide data support for practical interventions targeting these three ethnic groups and offer potential scientific and practical value for cross-cultural research on children’s executive function worldwide.

### 4.3 Limitation and future directions

Our study has some limitations. First, Briceño et al. (2026) cautioned that ethnic differences in cognitive function may be confounded by measurement differences. In the present study, while the assessments were administered by two research assistants fluent in the participants’ native languages, potential variability among the assistants cannot be entirely ruled out. Second, neither the WCST nor the Listening Sentence Span Task has been validated for use with Chinese ethnic minority children. The pilot study confirmed only that children in all three groups could understand and complete the tasks; no formal reliability, validity, or measurement-invariance testing was conducted. This may have introduced measurement bias into the observed group differences. Future studies should examine whether these tasks are validated among Chinese ethnic minority children. Third, we did not directly measure cultural beliefs, childrearing practices, parental educational involvement, language proficiency, socioeconomic status, or preschool quality. Future studies should directly measure family practices, parental educational involvement, language proficiency, socioeconomic status, and preschool quality, and test these as mediators or moderators of ethnic-group differences in EF. Fourth, the cross-sectional design limits the ability to infer causal relationships or track developmental trajectories over time. Subsequent studies could adopt longitudinal research methods to more accurately characterize changes in the developmental patterns of executive function among three ethnic groups. Finally, the present sample (N = 190; Yi children: 55; Bai children: 65; Na xi children: 70) fell marginally short of the 197 participants required to detect a medium ethnicity × age interaction (*f* = 0.025) withα = 0 0.05 and power = 0.80 in G*Power 3.1, although it exceeded the requirement of 158 for the main effects. Future studies should therefore recruit larger samples, and in particular more Yi children, who formed the smallest group, to further verify ethnic differences in executive function and their developmental trajectories.

## 5 Conclusion

Our study suggests that:(1) The pattern of executive function in Yi, Bai, and Naxi children aged 4 to 6 years showed commonality, which may be consistent with cultural universality. At the same time, the three ethnic groups exhibited certain differences in their developmental processes, the ranking of overall performance, from highest to lowest, was Bai children, Naxi children, and Yi children. (2) The developmental trajectories of different executive function components were not fully synchronized across the three ethnic groups. For inhibition control, the age of 5 appeared to be a period of relatively rapid development, and by age 6, inhibition control had largely matured. Cognitive flexibility showed a trend of relatively rapid developmental trend between ages 5 and 6. For working memory, at ages 4 and 6, the rank order of scores was Bai children, followed by Naxi children, and then Yi children; at age 5, Bai children scored significantly higher than both Naxi and Yi children. (3) Significant differences in cognitive flexibility were observed among Yi, Bai, and Naxi children aged 4 to 6 years, with performance ranking Bai children highest, followed by Naxi children, and Yi children lowest. Regarding pass rates on the Wisconsin Card Sorting Test (WCST), the rank order was Naxi children, followed by Bai children, and then Yi children at both ages 4 and 5; at age 6, however, the ordering shifted to Bai children, followed by Naxi children, and then Yi children.

## Data Availability

The data analyzed in this study is subject to the following licenses/restrictions: the datasets generated during this study are available from the corresponding author when the research topic is similar with the study. Requests to access these datasets should be directed to QL, quan_eng@163.com.
